# Adhesions due to peritoneal carcinomatosis caused by a renal carcinoma leading to mechanical gastric outlet obstruction: a case report

**DOI:** 10.1186/1752-1947-5-306

**Published:** 2011-07-13

**Authors:** Filippo Mocciaro, Gabriele Curcio, Ilaria Tarantino, Luca Barresi, Gaetano Burgio, Salvatore Gruttadauria, Settimo Caruso, Mario Traina

**Affiliations:** 1 Gastroenterology Unit, IsMeTT, UPMC, Via Tricomi 1, Palermo 90100, Italy; 2 Intensive Care Unit, IsMeTT, UPMC, Via Tricomi 1, Palermo 90100, Italy; 3 Surgery Unit, IsMeTT, UPMC, Via Tricomi 1, Palermo 90100, Italy; 4 Radiology Unit, IsMeTT, UPMC, Via Tricomi 1, Palermo 90100, Italy

## Abstract

**Introduction:**

Gastric outlet obstruction is a clinical syndrome caused by a variety of mechanical obstructions. Peptic ulcer disease used to be responsible for most gastric outlet obstruction, but in the last 40 years the prevalence of malignant tumors has risen significantly. Adhesive disease is an infrequent and insidious cause of mechanical gastric outlet obstruction.

**Case presentation:**

We report the case of a 78-year-old Caucasian man who had a clinical history of a right nephrectomy for malignancy three years earlier and who was admitted for a severe gastric outlet obstruction (score of 1) confirmed both by an upper endoscopy and by a fluoroscopic view after contrast injection. A computed tomography scan and a laparotomy, with omental biopsies, showed a peritoneal carcinomatosis with the development of abdominal adhesions that prompted an abnormal gastric rotation around the perpendicular axis of his antrum with a dislocation in the empty space of his right kidney. Symptoms disappeared after surgical bypass through a gastrojejunostomy.

**Conclusions:**

Our patient experienced a very rare complication characterized by the development of adhesions due to peritoneal carcinomatosis caused by a renal carcinoma treated with nephrectomy. These adhesions prompted an abnormal dislocation of his antrum, as an internal hernia, in the empty space of his right kidney.

## Introduction

Gastric outlet obstruction (GOO) is a clinical syndrome caused by a variety of mechanical obstructions (for example, malignancy, peptic ulcer disease, Crohn disease, and chronic pancreatitis). GOO is typically characterized by epigastric abdominal pain, early post-prandial vomiting with or without nausea, and weight loss. Before 1970, peptic ulcer disease was responsible for most GOO, but since the introduction of proton pump inhibitors in clinical practice 40 years ago, the prevalence of malignant tumors as the cause of GOO has risen to between 50% and 80% of all cases [1]. Adhesive disease from previous surgery is an infrequent cause of GOO but is a common cause of small bowel obstructions [2].

## Case presentation

**Figure 1:**
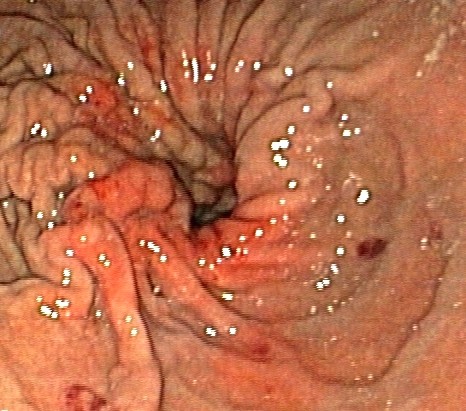
**Narrowing of lumen at upper endoscopy**.

**Figure 2:**
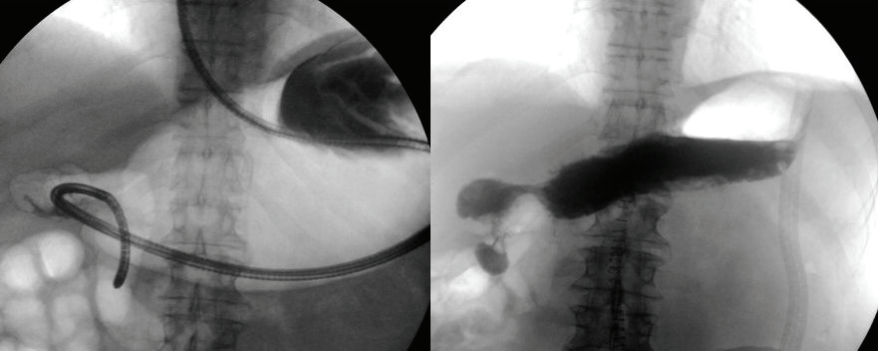
**Fluoroscopic view shows angulations of the antrum before and after contrast injection through a scope**.

**Figure 3:**
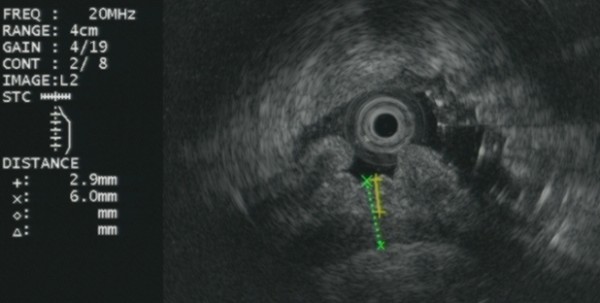
**Endoscopic ultrasound shows mild thickening of the mucosa with normal stratification of the gastric wall**.

**Figure 4:**
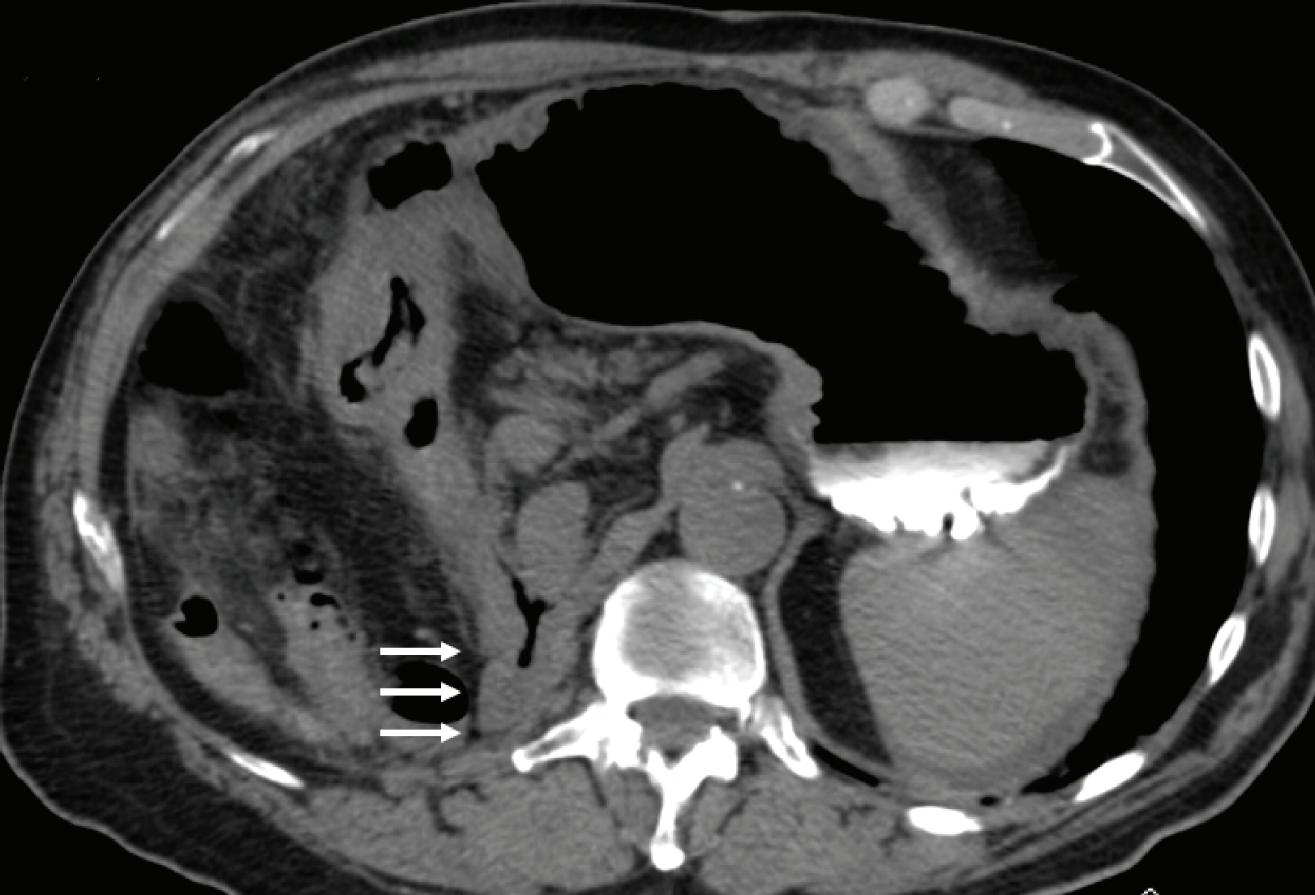
**Multi-detector computed tomography (MDCT) multi-planar reconstruction shows herniation of the duodenum into the renal space (white arrows)**.

A 78-year-old Caucasian man, referred to our institute by another hospital, was examined in our out-patient clinic for frequent episodes of post-prandial vomiting in the previous 30 days. The hospital referred him with a clinical and endoscopical suspicion of gastric lymphoma (severe stricture of his gastric antrum), although the results of his biopsy analysis were negative. A computed tomography scan confirmed the findings seen on upper endoscopy but offered no clear explanation of its nature. His clinical history included a right nephrectomy for malignancy three years earlier, although he underwent no chemotherapy. At examination, he appeared thin and malnourished and had a Gastric Outlet Obstruction Scoring System (GOOSS) score of 1 (0 = no oral intake, 1 = liquids only, 2 = soft foods, and 3 = solid food/full diet) [3]. His blood pressure, heart rate, and blood cell count were normal. His serum creatinine was high, although his electrolytes were within the normal range. No other significantly abnormal serum values were observed. We decided, on the basis of this evidence, to repeat the upper endoscopy in order to evaluate the stricture. His stomach appeared normal except in the corpus-antrum region, where his mucosa seemed congested in a significant narrowing of his lumen (Figure 1). The duodenum cannulation was difficult because of severe angulations of his antrum, which were confirmed by fluoroscopic view after contrast injection through the scope (Figure 2). At endoscopic ultrasound, performed with a 20 MHz UM-3R radial scanning ultrasonic miniprobe (Olympus Corporation, Tokyo, Japan) inserted in a therapeutic gastroscope (GIF-1TQ160; Olympus America Inc., Melville, NY, USA), the narrowed area appeared with mild thickening of his mucosa but with normal stratification of his gastric wall (Figure 3). All of his biopsy results were negative on pathological analysis. On a planned computed tomography scan, the bulb and the second portion of his duodenum appeared raised and inclined back toward his residual right kidney area (Figure 4). Widespread involvement of his peritoneum with irregular and nodular thickening was also observed. To resolve the GOO and obtain large omental biopsies, it was decided, in agreement with the surgeon, that our patient undergo a laparotomy with surgical bypass through a gastrojejunostomy. On biopsy, the final diagnosis of the pathologist was poorly differentiated omental carcinomatosis, probably related to the previous right renal carcinoma. Seven days after the operation, our patient's status was good, with regular transit through the gastrojejunostomy at fluoroscopy. He restarted oral feeding (GOOSS score = 3) without vomiting or other symptoms and, according to the oncologist, started chemotherapy for carcinomatosis.

## Discussion

Symptomatic adhesions after surgery are frequent (25% of readmissions in the first post-operative year) [2], and the risks increase considerably in the presence of peritoneal carcinomatosis [4]. However, adhesive disease can serve as an axis for gastric rotation around the long or the perpendicular axis of the stomach.

To the best of our knowledge, no data on the development of post-nephrectomy adhesions in patients operated on for renal malignancy have been published. In a 10-year study of 871 living-donor nephrectomies, less than 1% of patients experienced major complications and a mere 8% developed minor complications. There were no reports of adhesive disease [5]. A recent meta-analysis on laparoscopic versus open live-donor nephrectomy showed that laparoscopy is safer and found no development of adhesive disease after either type of surgery [6]. There is an interesting case report on an internal hernia in the retroperitoneum at the site of a previous nephrectomy in a 25-year-old living donor who developed signs and symptoms of partial small bowel obstruction [7].

In the long-term post-nephrectomy follow-up of patients with renal malignancy, the major concern is metastatic disease. The greatest risk of recurrence following resection for renal cell carcinoma is within three to five years after the operation, with predominant lung, bone, liver, brain, and local-regional involvement [8]. However, recurrence can occur anywhere, including the peritoneum, even if it is largely reported to be a consequence of ovarian, colonic, or hepatic malignancies. It is associated with a poor prognosis, limited treatment [9], and the development of adhesions with obstructive symptoms [4].

Our patient experienced a very rare complication characterized by the development of adhesions due to peritoneal carcinomatosis caused by a previous renal carcinoma treated with nephrectomy but not chemotherapy. These adhesions prompted an abnormal gastric rotation around the perpendicular axis of his antrum, with a dislocation, as an internal hernia, in the empty space of his right kidney. This case is interesting for two reasons: (a) GOO can occur as a late adhesive complication after abdominal surgery or peritoneal carcinomatosis or both, and (b) despite the low frequency of incidence, a late metastasis from renal carcinoma can involve the peritoneum without ascites but with severe obstructive symptoms.

## Conclusions

This report highlights the importance of regular out-patient visits in patients with a history of neoplasms, even if they have undergone surgery and especially if they have not been treated with chemotherapy. Particular attention should be paid to new obstructive symptoms as possible consequences of late post-surgical or unusual peritoneal metastatic complications.

## Abbreviations

GOO: gastric outlet obstruction; GOOSS: Gastric Outlet Obstruction Scoring System.

## Consent

Written informed consent was obtained from the patient for publication of this case report and any accompanying images. A copy of the written consent is available for review by the Editor-in-Chief of this journal.

## Competing interests

The authors declare that they have no competing interests.

## Authors' contributions

FM collected the data and wrote the article. GC, IT, and LB were involved in drafting the manuscript and revising it critically for important intellectual content. GB, SG, and SC were involved in revising the manuscript critically for important intellectual content. MT was involved in revising the manuscript critically for important intellectual content and gave final approval of the version to be published. All authors read and approved the final manuscript.
